# Non-Viral Nucleic Acid Delivery Strategies to the Central Nervous System

**DOI:** 10.3389/fnmol.2016.00108

**Published:** 2016-11-01

**Authors:** James-Kevin Y. Tan, Drew L. Sellers, Binhan Pham, Suzie H. Pun, Philip J. Horner

**Affiliations:** ^1^Department of Bioengineering and Molecular Engineering & Sciences Institute, University of WashingtonSeattle, WA, USA; ^2^Center for Neuroregenerative Medicine, Houston Methodist Research InstituteHouston, TX, USA

**Keywords:** central nervous system, delivery, *in vivo*, non-viral, nucleic acid

## Abstract

With an increased prevalence and understanding of central nervous system (CNS) injuries and neurological disorders, nucleic acid therapies are gaining promise as a way to regenerate lost neurons or halt disease progression. While more viral vectors have been used clinically as tools for gene delivery, non-viral vectors are gaining interest due to lower safety concerns and the ability to deliver all types of nucleic acids. Nevertheless, there are still a number of barriers to nucleic acid delivery. In this focused review, we explore the *in vivo* challenges hindering non-viral nucleic acid delivery to the CNS and the strategies and vehicles used to overcome them. Advantages and disadvantages of different routes of administration including: systemic injection, cerebrospinal fluid injection, intraparenchymal injection and peripheral administration are discussed. Non-viral vehicles and treatment strategies that have overcome delivery barriers and demonstrated *in vivo* gene transfer to the CNS are presented. These approaches can be used as guidelines in developing synthetic gene delivery vectors for CNS applications and will ultimately bring non-viral vectors closer to clinical application.

## Introduction

The incidence of neurological diseases and injuries is increasing with the rising life expectancy (Mattson and Magnus, 2006). Nucleic acid therapeutics, such as genes and small interfering RNA (siRNA) oligonucleotides have emerged as a promising treatment strategy to preserve neuron function, enhance neurogenesis and prevent the progression of neurological diseases. The delivery of nucleic acids encoding brain-derived neurotrophic factor (Huang et al., 2012a), epidermal growth factor (Sugiura et al., 2005), fibroblast growth factor-2 (Matsuoka et al., 2003), Huntingtin (Burgess et al., 2012), neurogenin-2 (Zhang et al., 2013; Masserdotti et al., 2015), insulin growth factor-1 (Kaspar et al., 2003), and vascular endothelial growth factor (Dodge et al., 2010) have been shown to increase neuron regeneration or delay the progression of neurological diseases in mice, rats and gerbils. Targeting gene delivery vehicles to the appropriate cells and proper protein regulation remain the primary challenges to making these pathways feasible. While viral vectors such as the adeno-associated virus have typically been used clinically, interest in non-viral nucleic acid delivery remains high due to lower safety concerns, greater customizability and an ease in manufacturing (Pack et al., 2005; Burke et al., 2013). In fact, the number of synthetic vectors used in gene therapy clinical trials has been steadily increasing over the last 10 years (Gene Therapy Clinical Trials Worldwide, Wiley).

With neurological diseases specifically affecting different parts of the brain and even sub-phenotypes of neural cells, the route of administration is a crucial aspect of nucleic acid delivery. Intraventricular injection places therapeutics closer to the subventricular zone, one of the stem cell niches of the brain, whereas localized intraparenchymal injections may be used to target a specific part of the brain where neurodegeneration is occurring or at the location of disease (e.g., brain tumor). In this focused review, we explore the barriers facing *in vivo* nucleic acid delivery and highlight the recent synthetic vehicles and different strategies that have overcome these challenges to deliver nucleic acids to the central nervous system (CNS). First, we briefly discuss nucleic acid protection and targeting the CNS since these strategies apply to any route of administration. In later sections, we discuss the common routes of administration and the specific barriers and vehicular solutions accompanying each method. While a wide variety of delivery vehicles have been applied to nucleic acid CNS delivery, we primarily focused on lipidic and polymeric vehicles with a few selected examples of inorganic delivery vehicles.

### Nucleic Acid Protection

**Figure 1:**
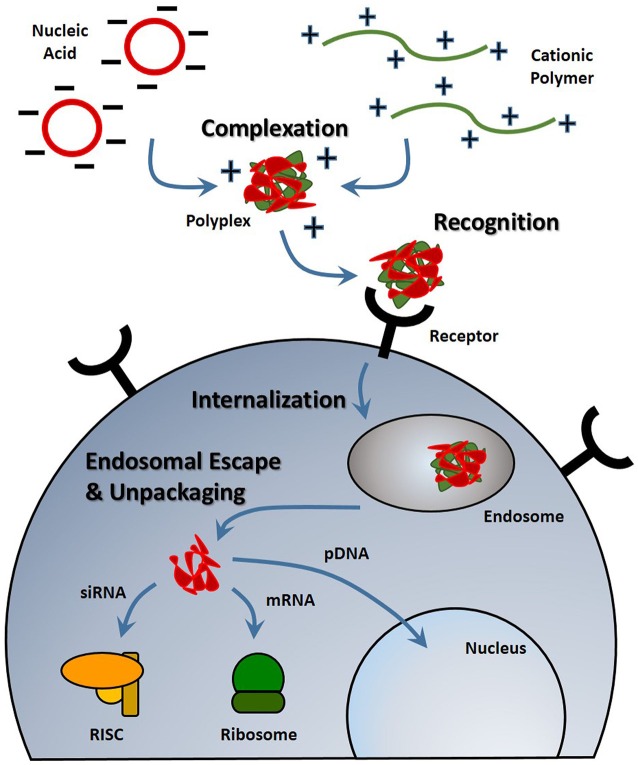
**Stages of nucleic acid delivery into a cell.** Nucleic acids are typically condensed and complexed with a cationic material. This complex must be recognized by a cell, be internalized and escape the endosomal-lysosomal degradation pathway. Once in the cell cytoplasm, the nucleic acid can separate from its vehicle and traffic to its intended target based on its type.

With any route of administration, nucleic acids are susceptible to chemical degradation and clearance from the body due to the presence of extracellular nucleases and the immune system (Abdelhady et al., 2003). While naked nucleic acid delivery is feasible, carrier-mediated delivery has the potential to be more efficient by protecting nucleic acids and chaperoning nucleic acids through the extracellular and cellular barriers to gene delivery (Figure 1). Thus, this focused review focused on carrier-mediated delivery of nucleic acids. Typically, negatively charged nucleic acids are complexed and condensed with cationic, synthetic materials which allows for nucleic acids to remain hidden and avoid degradation (Pack et al., 2005). Common complexation agents used for *in vitro* gene transfer include cationic polymers such as polyethylenimine (PEI), which electrostatically bind to nucleic acids to form “polyplexes,” or polymer-nucleic acid complexes. Similarly, cationic lipids can also be used to complex nucleic acids to form “lipoplexes”. Another method of enhancing stability is by modifying the nucleic acid itself so that it avoids recognition and degradation. For example, altering the ribose moiety and introducing 2′-fluoro and phosphorothioate near the terminal region of siRNA duplexes enhanced stability and prolonged siRNA half-life *in vivo* (Wang et al., 2008).

Despite the protection afforded to nucleic acids by electrostatic complexation, these cationic complexes are still subject to challenges such as aggregation, toxicity, premature sequestration by phagocytic cells, and non-specific interaction with cell membranes and serum proteins (Morille et al., 2008). When intravenously administered, PEI, one of the most effective transfection agents *in vitro*, causes severe toxic side effects due to polyplex aggregation and strong electrostatic interactions with cell membranes, proteins and the extracellular matrix (Al-Dosari and Gao, 2009). To overcome these challenges, shielding strategies have been developed to hide nucleic acid delivery vehicle by moieties like poly(ethylene glycol) (PEG) and albumin (Lu et al., 2006; Laga et al., 2012).

PEG, a biocompatible, hydrophilic polymer, is commonly used as a shielding agent for nanoparticles (Immordino et al., 2006). By creating a barrier around the complex, it was previously believed that PEG prevents the adsorption of proteins (Pombo García et al., 2014). However, recent studies suggest that PEGylation of nanoparticles results in preferential binding of clusterin, a chaperone protein that binds to hydrophobic domains of unfolded proteins and prevents non-specific binding (Schöttler et al., 2016). When conjugated to delivery vehicles, PEG has been shown to improve the stability and increase the circulation half-life of polyplexes, liposomes and other types of vectors. For example, when conjugated to PEI, PEG prevented the aggregation of PEI-plasmid DNA complexes in fetal bovine serum-enhanced media, resulting in complexes that could circulate long enough for observed localization in the brain (Son et al., 2011). With encapsulation in PEGylated liposomes, PEI-oligonucleotide complexes were able to circulate substantially longer and had plasma concentrations significantly higher than naked complexes after 60 min (Ko et al., 2009). By any route of administration, protecting delivery vehicles with a shielding agent minimizes aggregation and premature sequestration and can increase the distribution of the nucleic acids to desired cells.

### Targeting the CNS and Neuronal Cells

Nevertheless, prolonged stability and circulation is not sufficient for substantial nucleic acid delivery to the brain and spinal cord because the nervous system is protected by barriers that grossly prevent the access of therapeutics (Barchet and Amiji, 2009). Cells of the CNS can potentially be accessed through several contact points including: (1) the blood system; (2) the cerebral spinal fluid (CSF) in the ventricles or lumbar space; (3) intraparenchymal fluid in the extracellular space; or (4) nerve endings that extend outside of the nervous system (Cipolla, 2009). The nervous system is sequestered behind a barrier system composed of vascular tight junctions and glial elements that ensheath the blood supply producing a blood-brain and blood-spinal cord barrier (BBB and BSCB, respectively; Banks, 2016). Each of these compartments represent a potential entry point as well as unique challenges for neuronal targeting. Vehicles for systemic delivery must utilize a mechanism that will facilitate penetration and uptake across the BBB or BSCB (Spencer and Verma, 2007; Tobinick, 2016). Within the brain, paracellular flow of neuro-active cytokines is controlled by pulsation of the blood vessels that mechanically drives a peristaltic movement of extracellular fluid (Johanson et al., 2011; Iliff et al., 2013). Meanwhile, gene delivery vehicles directly administered into the ventricles need to bind to cells of interest before being washed out of the CNS (Syková and Nicholson, 2008). At the periphery, a vehicle that promotes uptake at nerve termini and retrograde transport must be able to target a neuron for transfection and promote travel along the neuronal cytoskeleleton into the CNS (Hanz and Fainzilber, 2004; von Bartheld, 2004; Medina-Kauwe, 2007; Tarragó-Trani and Storrie, 2007). Consequently, the advent and development of targeting ligands has greatly enhanced the capacity of non-viral vectors to deliver nucleic acids into the CNS.

Drug delivery vehicles have been modified with targeting agents such as peptides, antibodies, proteins and sugars to specifically home therapeutics to desired tissues and cell types. For systemic administration, active targeting is important in directing the accumulation of vehicles at the brain endothelium. One commonly used brain-targeting molecule is transferrin, a glycoprotein that binds to iron. Transferrin receptor is expressed on the brain endothelium and the binding of transferrin-decorated nucleic acid delivery vehicles to these receptors allows for accumulation right outside the brain (Huang et al., 2007).

Vehicles administered directly into brain by intraventricular or intraparenchymal methods can also benefit from active targeting by directing the delivery of nucleic acids to pertinent cells. For example, to more specifically transfect neural progenitor cells, Tet 1, a peptide that specifically binds to neuronal cells, was conjugated to PEI complexes (Kwon et al., 2010). This Tet1-PEI polymer led to a significantly improved transfection of neural progenitors by targeted complexes over untargeted complexes. Thus, targeting moieties can help deliver nucleic acids to cells and tissues of interest while minimizing non-specific delivery. In addition, targeting ligands can improve the intracellular delivery of nucleic acids since many targeting ligands are endocytosed by cells after binding to its receptor. Decorated macromolecules such as polyplexes and liposomes show enhanced uptake in cells compared to their non-targeted counterparts.

## Systemic Delivery

Intravenous administration is one of the most common routes of administration for macromolecule therapeutics such as nucleic acids and has the advantage of rapid distribution and high bioavailability. However, systemic circulation presents a major challenge for nucleic acid delivery. Naked DNA has poor stability and is rapidly broken down by nucleases, sequestered by the liver, and cleared from circulation with a plasma half-life of mere minutes (Emlen and Mannik, 1984; Kawabata et al., 1995). To prevent premature degradation and prolong circulation, nucleic acids have been complexed with PEGylated cationic materials, such as polymers and liposomes, which act to shield the polyplex and facilitate compact packaging and protection. Targeting ligands conjugated to the synthetic vectors can facilitate recognition of brain endothelium. However, transport into the brain requires crossing the BBB, a tight network of endothelial cells that restricts entry into the brain parenchyma (Gabathuler, 2010). The brain endothelium has a high expression of efflux pumps and transporter proteins that exclude nearly 100% of large-molecule therapeutics and more than 98% of all small-molecule drugs (Begley, 2003; Pardridge, 2005). Recent *in vivo* investigations have focused on transportation across the BBB and temporarily disrupting the BBB after systemic administration.

### Transport Across the Blood-Brain Barrier

Strategic selection of brain targeting ligands can result in both recognition of the brain endothelium and facilitated transcytosis across the BBB. This process, called receptor-mediated transcytosis, has been demonstrated with cationic proteins and is believed to be carried out by clathrin-coated pits or caveolae (Hervé et al., 2008; Table 1). After these materials bind to the luminal surface of the brain endothelial cells, vesicular transcytosis is mediated by different proteins and the high concentration of mitochondria in endothelial cells to cause exocytosis at the abluminal surface.

**Table 1:** **Properties of effective nucleic acid delivery vehicles**.

Property	Function	Material examples	Schematic
Nucleic acid packaging	Condense, package, and protect DNA, RNA, or siRNA	PEI, PLL, PAMAM, liposomes	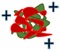
Stability	Prevent premature unpackaging and avoid sequestration and clearance	PEG, albumin	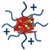
Targeting	CNS localization and cell-specific uptake	Peptides, antibodies, proteins	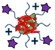
Endosomal escape	Facilitate release from the endosome to avoid lysosomal degradation	Melittin, pH sensitive materials, amines for proton sponge effect	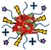
Cargo release	Triggered release or detachment from nucleic acid	Disulfide linkages	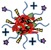

The transferrin receptor is frequently targeted for BBB transcytosis. After binding the transferrin receptor carrier protein, the transferrin-iron complex is internalized at the apical side of the brain endothelium and is eventually exocytosed at the opposite basal surface. Since transferrin receptor is expressed on the BBB and transcytoses transferrin, it can be utilized as an uptake pathway into the brain by nucleic acid vehicles functionalized with transferrin. In one example, polyamidoamine (PAMAM) dendrimers were decorated with transferrin by a PEG linker and showed a ~2-fold higher brain uptake and gene transfer compared to PEGylated dendrimers alone (Huang et al., 2007). In another example, transferrin antibodies were used to decorate liposomes that hid plasmid DNA. This system was able to show a 10-fold greater β-glucuronidase enzyme activity in murine brains deficient of the protein (Zhang et al., 2008).

Another BBB transcytosis moiety is the rabies viral glycoprotein (RVG) peptide, a 29-amino acid peptide which binds to nicotinic acetylcholine receptors. Modifying the peptide sequence to include nine arginines on the C-terminus allows for complexation with nucleic acids (Kumar et al., 2007). Upon systemic intravenous administration, these complexes were able to transvascularly deliver siRNA to the brain through clathrin- and caveolae-mediated endocytosis by endothelial cells, which lead to extended lives of encephalitic mice. The RVG peptide can also modulate the accumulation of larger vehicles and has delivered macro-structures such as PAMAM dendrimers (Liu et al., 2009), liposomes (Pulford et al., 2010), chitosan nanoparticles (Gao et al., 2014), poly(mannitol-*co*-PEI) complexes (Park et al., 2015) and exosomes (Alvarez-Erviti et al., 2011) across the BBB and into the brain parenchyma. Other targeting agents have included: angiopep, a peptide that binds to low-density lipoprotein receptor-related protein-1 (Ke et al., 2009); lactoferrin, an iron-binding protein of the transferrin family (Huang et al., 2008, 2010); leptin, a peptide that binds to leptin receptor in different parts of the brain (Liu et al., 2010); chlorotoxin, a scorpion-derived venom that is a specific marker for gliomas (Costa et al., 2013); TGN peptide, a BBB targeting peptide isolated by phage display (Qian et al., 2013); and LIMK2 NoLs peptide, a nucleolar translocation signal sequence derived from the LIM Kinase 2 protein (Yao et al., 2015). Collectively, these targeting agents have shown to facilitate the accumulation of PEGylated PAMAM dendrimers, lysine dendrimers, liposomes, and polymeric polyplexes in the brain.

### Blood-Brain Barrier Disruption

Other methods of systemic nucleic acid delivery focus on temporarily disrupting the BBB to enhance the diffusion of vehicles into the brain. These strategies can be combined with delivery vehicles to further augment gene transfection. Small molecules, such as mannitol, have been shown to temporarily open the BBB and allow the penetration of larger molecules into the brain parenchyma. Hypertonic solutions of these molecules is believed to widen tight junctions by shrinking vascular endothelial cells (Rapoport, 2001). Consequently, the co-administration of mannitol with RVG-decorated PEI was able to significantly enhance the distribution of complexes throughout the brain when compared to carriers alone (Hwang et al., 2011).

**Figure 2:**
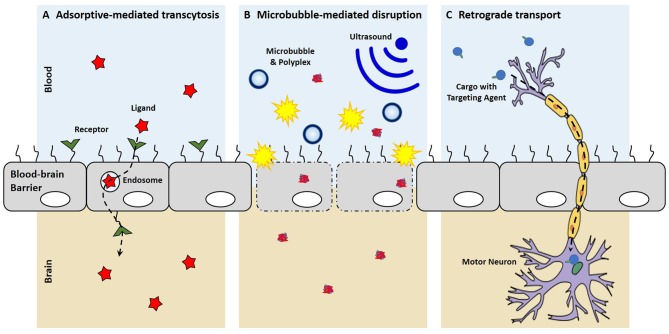
**Mechanisms of entering the central nervous system (CNS). (A)** With receptor-mediated endocytosis, the binding of a ligand to its receptor on the brain endothelium facilitates cellular endocytosis, vesicular trafficking and eventually exocytosis on the contralateral side into the brain. **(B)** Microbubble-mediated disruption of the choroid plexus epithelium breaks tight junctions and creates micropores, allowing for the enhanced penetration of polyplexes into the brain. **(C)** New targeting ligands allow for uptake by peripheral neurons and the retrograde transport of cargo along axons to cell bodies in the CNS.

More recently microbubbles, or gas-filled microspheres, have been coupled with ultrasound as a method to temporarily disrupt the BBB (Meairs and Alonso, 2007; Panje et al., 2013; Rychak and Klibanov, 2014; Figure 2). This process, called sonoporation, creates micropores, permeabilizes cell membranes and breaks up tight junctions as microbubbles act as local enhancers of the ultrasound acoustic energy and cavitate causing local shear flow, microstreams and microjets (Greenleaf et al., 1998; Zhou et al., 2012; Panje et al., 2013). These BBB openings are large enough to allow for the permeation of macromolecules into the brain such as immunoglobulin G and 70 kDa dextran (Sheikov et al., 2004; Choi et al., 2007, 2010, 2011; Xie et al., 2008). The safety of microbubble-mediated BBB disruption has been evaluated in rats and macaque monkeys with no or limited damage to brain tissue and no behavioral or visual deficits (McDannold et al., 2012; Kobus et al., 2015). Microbubble-mediated disruption of the BBB has been used to increase anti-Huntingtin siRNA delivery into the murine brain to reduce Huntingtin protein levels in animal disease models (Burgess et al., 2012). In another example, DNA can be complexed with cationic polymer-decorated microbubbles to prevent premature degradation. In this manner, microbubble and DNA complexes were used to markedly enhance the expression of brain-derived neurotrophic factor and enhanced green fluorescence protein in murine brains (Huang et al., 2012a,b). Microbubbles can also be co-administered with liposomal gene carriers to specifically open a region of the BBB by focused US, which uses an acoustic lens to center the US at a specific point (Lin et al., 2015). Co-administration of microbubbles with dense PEG-coated nanoparticles was able to open the BBB and transfect a variety of brain cells for at least 28 days with systemic administration (Nance et al., 2014; Mead et al., 2016).

In some cases, such as brain gliomas and traumatic brain injury, there is a natural disruption of the BBB. In small rat brain tumors, there is no BBB permeability; however, as the tumor grows and neovascularization occurs, ultrastructural defects in the capillary vessels arise causing a stark disruption in the tumor (Yamada et al., 1982). This leaky BBB vasculature can be exploited by gene delivery vehicles as a facile method of CNS entry and can greatly complement receptor-mediated transcytosis. PEGylated nanoparticles and liposomes decorated with receptor-mediated transcytosis ligands were able to accumulate in gliomas after systemic administration and delay tumor growth (Lu et al., 2006; Yue et al., 2014).

## Cerebrospinal Fluid Injection

The CSF is produced by the choroid plexus deep in the ventricles and drains along paravenous circulation (Cipolla, 2009). The CNS is unique in that it does not have a traditional lymph system and waste material and metabolites drain from the extracellular space to the CSF (Iliff et al., 2012). While non-viral gene delivery into CSF-filled spaces is not as facile as intravenous administration, it does have the advantages of avoiding systemic circulation and placing therapeutics in close proximity to the brain parenchyma (Johanson et al., 2011). For example, lateral intraventricular injections allow for close proximity to the subventricular zone, a region of the brain containing neural progenitor cells, and may be an appropriate injection site for neurogenesis applications. In addition, there are excellent access points that are used routinely in the clinic including a lumbar puncture or intraventricular cannulas (Belverud et al., 2008; Tobinick, 2016). Substances directly injected into the CSF circumvent the BBB and distribute in the brain depending on size and charge. The ependymal barriers in ventricles are comprised of the choroid plexus cells and in the sub-arachnoid space, the arachnoid barrier cells of glia and pial vessels (Abbott et al., 2006). For intraventricular delivery, large molecular weight proteins are not free to diffuse into the brain parenchyma due to the choroid plexus epithelium, which lines the cavities of the ventricles and the cranial and spinal sub-arachnoid space and secretes cerebrospinal fluid (Dohrmann, 1970; Mortazavi et al., 2014). While the ependymal barrier is not as stringent as the BBB, access and penetration into the brain parenchyma are still difficult due to the low diffusion and the constant movement of CSF fluid through the CNS and back into the bloodstream (Pardridge, 2011). While ventricular access of therapeutics to the brain is restricted, delivery to the sub-arachnoid space often results in widespread brain delivery of small molecular weight proteins (Iliff et al., 2012).

### Designing Vehicles to Overcome Delivery Barriers

While the choroid plexus ependymal cell layer acts as a barrier, nucleic acid therapeutics intraventricularly injected are still able to have some efficacy likely due to the direct sampling of neural stem cells into the CSF and some penetration into the parenchyma. Certain formulations of linear PEI were able to diffuse throughout the ventricular space and transfect neurons and glia near the edge (Goula et al., 1998). After intraventricular injection, PEI and DNA complexes have been shown to transfect neural progenitor cells (Lemkine et al., 2002); while PEI complexes decorated with Tet1, a peptide that specifically binds to neuronal cells, bound better to neural progenitor cells and showed an improved transfection of neural progenitors over untargeted complexes (Kwon et al., 2010). Other carrier structures such as liposomes and silica nanoparticles have also been used as nucleic acid delivery vehicles for intraventricular administration. Cationic liposomes and organically modified silica nanoparticles were used to successfully deliver siRNA and plasmid DNA to neuronal cells *in vivo*, respectively (Bharali et al., 2005; Zou et al., 2010). All of these carriers can help protect nucleic acids as lipoplexes protected mRNA from premature degradation in CSF for up to 4 h while mRNA alone degraded within 5 min (Anderson et al., 2004). Peptide-decorated micelles filled with dexamethasone, a glucocorticoid that facilitates transport to the nucleus, were able to significantly reduce infarct size after middle cerebral artery occlusion by gene delivery.

Recently, multifunctional gene delivery vehicles have been synthesized with the aim of overcoming many of the barriers to gene delivery such as premature unpackaging, endosomal escape, and DNA release (Table 1). A statistical copolymer of *N*-(2-hydroxypropyl)methacrylate (HPMA), oligo-*L*-lysine, and melittin was developed for gene delivery after intraventricular injection (Schellinger et al., 2013). The HPMA monomers were for stability, the lysines were developed for DNA condensation and packaging, and the melittin, a membrane-lytic peptide developed from honey bee venom, was included to enhance endosomal escape after vesicular uptake. This polymer efficiently condensed DNA into stable particles to form polyplexes and increased brain transfection about 35-fold compared to melittin-free analogs. Another polymer designed for *in vivo* gene delivery utilized a double-headed reversible addition-fragmentation chain transfer agent and a ring-opening polymerization initiator to create two different polymer segments that contribute to different aspects of gene delivery (Wei et al., 2013). This copolymer, PCL-SS-p[(GMA-TEPA)-*s*-OEGMA], consisted of a block of poly(ε-caprolactone) (PCL) connected by a reducible disulfide to a statistical copolymer of tetraethylenepentamine (TEPA)-decorated poly(glycidyl methacrylate) (GMA) and oligo(ethylene glycol) monomethyl ether methacrylate (OEGMA). The TEPA amine groups bind to and condense nucleic acids to form polyplexes while the hydrophobic PCL and hydrophilic OEGMA provide extracellular stability. After polyplex internalization, the amine groups contribute to endosomal escape by pH buffering and the internal disulfide bond can be reduced by cytosolic glutathione facilitating polyplex destabilization and nucleic acid release. These polyplexes were shown to have diameters less than 200 nm, transfected HeLa cells more efficiently than PEI *in vitro*, and delivered luciferase genes to the brain more efficiently than its individual components. To improve transfection further, the amines of this polymer were guanidinylated and investigated *in vivo*. The delocalized charge of guanidinium groups is attributed to stronger interaction with DNA than amines and greater cell internalization by interacting with cell surface phosphates and sulfates (Wehling et al., 1975; Cheng et al., 2013). While guanidinium groups show improved transfection *in vitro*, they did not translate to augmented transfection *in vivo* likely due to premature unpackaging as guanidinium groups have a predilection for sulfates of heparan sulfate proteoglycans over nucleic acid phosphate groups (Choi et al., 2015). Recently, we developed a new endosomal-escaping, polymeric vehicle that has a triggered exposure of a membrane lytic peptide when in the acidic pH of endosomes (Cheng et al., 2016). This polymer, called Virus-Inspired Polymer for Endosomal Release (VIPER), is composed of a cationic block, poly(OEGMA)-*co*-poly(2-(dimethylamino)ethyl methacrylate) (p(OEGMA-DMAEMA)), for nucleic acid condensation and a pH-sensitive block, poly(2-diisopropylaminoethyl methacrylate)-*co*-poly(pyridyl disulfide ethyl methacrylate) (p(DIPAMA-PDSEMA)), for triggered display of a membrane lytic peptide, melittin, in acidic conditions. VIPER polyplexes, or polymer-DNA complexes, showed membrane-lytic activity only in the acidic conditions of the cell endosome and efficient gene transfer to a variety of cell types and therefore may be useful for CNS gene transfer.

Cerebrospinal fluid injections into other areas of the CNS have also been employed for the administration of nucleic acid carriers. After injection into the cisterna magma of rats, liposomes delivered luciferase plasmid throughout the brain that was still detectable 7–10 days later (Hauck et al., 2008). Interestingly, when the same system was directly injected into the parenchyma, luciferase expression was not as distributed. A micelle system of PEG-aspartic acid polymer was able to provide sustained protein expression with minimal immunogenicity (Uchida et al., 2013). Nucleic acid administration into the lumbar subarachnoid space has also been accomplished with a variety of delivery vehicles. Poly(lactic-co-glycolic acid) microparticles containing plasmid DNA that encodes IL-10 were able to relieve neuropathic pain in rats for greater than 74 days (Soderquist et al., 2010). PEI complexes decorated with a peptide from nerve growth factor were able to more specifically transfect dorsal root ganglia (Zeng et al., 2007). By creating Tat decorated-PEI complexes with magnetic iron beads, researchers were able to use magnetic fields to direct the movement of DNA complexes into remote areas away from the injection site in rat spinal cords (Song et al., 2010). While sufficient levels of gene transfection are achieved after CSF injection, the choroid plexus epithelium still prevents most of the vehicles from entering the parenchyma which results in a significant loss of transfection potential.

### Choroid Plexus Epithelium Disruption

In a similar fashion to BBB disruption, the choroid plexus epithelium may be transiently disrupted by microbubbles and ultrasound to allow for the enhanced penetration of materials into the brain parenchyma from the CSF fluid. Custom microbubbles were prepared and did not aggregate with aforementioned PCL-SS-p[(GMA-TEPA)-*s*-OEGMA] polyplexes (Tan et al., 2016). In *in vitro* transwell assays, these microbubbles were able to sonoporate immortalized choroid plexus monolayers to allow for the enhanced flow through of 5 kDa PEG and 70 kDa dextran. Upon *in vivo* administration into murine ventricles, the microbubbles and ultrasound were able to significantly increase polyplex transfection of cells with luciferase compared to polyplexes alone or polyplexes and microbubbles without ultrasound. Temporary microbubble-mediated disruption of the choroid plexus epithelium seems like a viable strategy to enhance the penetration of polyplexes and may garner more research in the future.

## Intraparenchymal Injection

Intraparenchymal injection is the most direct access to discrete anatomy and cells of the brain and spinal cord. However, there are several critical challenges notwithstanding the inherent risk of an invasive CNS injection. Injection or probe placement alone can create a reactive gliosis that may limit the transport of a therapeutic or exacerbate the disease (Polikov et al., 2005; Potts et al., 2013). Methods for convection enhanced delivery and the use of small caliber pipettes can mitigate some of these concerns and allow targeted delivery of relatively large volumes without harm (Mano et al., 2016). Once in the parenchyma, therapeutics have variable degrees of diffusion and entrapment that can be modeled based on protein or drug size and composition (Patlak et al., 1983; Ghersi-Egea et al., 2002; Hrabe et al., 2004; Nicholson et al., 2011). After parenchymal diffusion, clearance is regulated by the lymphatic system which is comprised of the glia cells that ensheath the venous system of the brain which is localized primarily near the dural surfaces (Iliff et al., 2012; Louveau et al., 2015). In general, small molecular weight substances are able to diffuse readily and are cleared in minutes. Larger molecular weight substances may either lack significant diffusion or be cleared over a course of hours (Syková and Nicholson, 2008).

### Bolus Injection

While the location of intraparenchymal injections into the brain can vary, the same type of nucleic acid delivery vehicles is still utilized. PEI decorated with 2 kDa PEG chains resulted in improved gene delivery after intrathecal administration into the lumber spinal cord subarachnoid space compared to PEI (Tang et al., 2003). Reducible arginine-PAMAM dendrimers were able to knockdown genes after injection into the cortex (Kim et al., 2010). Biodegradable poly(*β*-amino esters) that were lyophilized and stored for over 2 years effectively transfected brain glioblastomas, demonstrating long-term storage and efficacy for clinical translation (Guerrero-Cázares et al., 2014). Reversibly conjugated siRNA to liposomes was able to efficiently silence genes in oligodendrocytes after administration into the corpus callosum (Chen et al., 2010). Liposomes encapsulating siRNA have also shown effective gene knockdown of the GluN1 subunit of NMDA receptors in neurons (Rungta et al., 2013). Targeting agents have also been used to enhance the nucleic acid delivery vehicles. PEGylated PEI was targeted with folate, which binds to folate receptor often overexpressed on cancer cells, and liposomes were targeted with transferrin to improve the delivery of plasmids and siRNA after injection into the right striatum (Cardoso et al., 2008; Liang et al., 2009).

### Sustained Delivery

The compact and tortuous morphology of the brain parenchyma severely limits the diffusion of nucleic acid delivery vehicles away from the administration site. To overcome this, sustained delivery is utilized to constantly introduce more vehicles and increase the diffusion throughout the brain. In one example, an osmotic pump was able to continually inject siRNA and liposome complexes into the frontal lobe to knockdown the resistance of gliomas to therapy (Kato et al., 2010). This treatment significantly sensitized tumors to the chemotherapeutic agents and extended the survival of mice. In other cases, a cannula is implanted in the brain for acute direct injections or chronic administration. Repeated dosing of siRNA against toxic Huntingtin protein in *β*-cyclodextin carriers was able to alleviate motor deficits in a Huntington’s disease mouse model (Godinho et al., 2013).

Like the osmotic pump, convection-enhanced delivery is administered intraparenchymally and used to continually introduce therapeutics. A cannula is typically inserted stereotaxically into a designated spot in the brain and a therapeutic fluid is continuously injected under positive pressure (Allard et al., 2009). The administration of siRNA by convection-enhanced delivery was able to silence genes in oligodendrocytes (Querbes et al., 2009) and silence Huntingtin gene in a widespread manner across the brain (Stiles et al., 2012). When a cell-penetrating peptide, TAT, was attached to liposomes, gene transfection increased *in vivo*; however, expression was restricted to the vicinity of the infusion catheter (MacKay et al., 2008). When comparing positively and negatively charged liposomes, anionic liposomes were better able to spread throughout the brain parenchyma with similar transfection levels (Kenny et al., 2013).

## Retrograde Transport

While there have been substantial advances in brain-targeted delivery to treat diseases that affect specific parts of the brain like Alzheimer’s Disease (Kumar et al., 2007; Spencer and Verma, 2007; Yu et al., 2011), few therapeutic options are available for degenerative diseases that affect motor neurons because many of the potential genes and siRNA drugs show limited diffusion and penetration to motor neurons deep in the CNS parenchyma (Monani, 2005; Mitchell and Borasio, 2007). For decades, classes of viruses have been known to infect neuronal projections in the periphery and undergo retrograde axonal transport into the brain and spinal cord (LaVail and LaVail, 1972; Salinas et al., 2010). Thus, several lab have begun to systematically mutate adeno-associated vectors in order to expand their clinical application and increase delivery into the CNS (Maheshri et al., 2006; Kotterman and Schaffer, 2014), innovative strategies have been adopted to utilize retrograde axonal transport to deliver biologics into the spinal cord (Xu et al., 2005; Hollis et al., 2008; Snyder et al., 2011). As a result, these viruses have been engineered and shown to be effective for remote gene transfer into the CNS after intramuscular injection to induce neurotrophic factor expression in animal models of neurodegenerative disease (Kaspar et al., 2003; Azzouz et al., 2004; Petruska et al., 2010; Benkhelifa-Ziyyat et al., 2013; Hirano et al., 2013).

Recently, small targeting agents have been used to direct the trafficking of cargo into the CNS after peripheral administration (Figure 2). Tetanus toxin subunit-C (TTC), an atoxic fragment of tetanus toxin that contains the ganglioside-binding site, is able to mediate uptake at both pre- and post-synaptic at nerve termini to allow retrograde transport passage of TTC within neurons (Price et al., 1975; Schwab et al., 1979). Consequently, these trans-synaptic properties of TTC have been exploited as a fusion protein to enable delivery into the spinal cord after TTC uptake at peripheral nerve termini (Francis et al., 2004; Chian et al., 2009; Li et al., 2009). While the TTC fusion-proteins were shown to increase delivery into the spinal cord, these studies were not able to discern a therapeutic benefit, which may suggest that that TTC-fusions do not escape the endosome after uptake and remain sequestered in the vescicle that mediated uptake. More recently, a targeted axonal import peptide (TAxI) was identified by *in vivo* phage display. The TAxI peptide was able to mediate uptake and delivery of an active Cre recombinase into the nucleus of spinal cord motor neurons after hind limb intramuscular injection (Sellers et al., 2016). These data suggest that small peptides are not only able to mediate synaptic uptake at nerve termini and retrograde transport within neurons, but they allow for functional protein cargo delivery via the neuron. While there have yet to be any reports of synthetic, retrograde nucleic acid delivery into the CNS, the discovery of targeting ligands that mediate uptake by neurons in the periphery, transport within neurons to the CNS, and release of active cargo into the cytoplasm has the potential of opening a whole new delivery route for non-viral technologies to target motor neurons for gene delivery.

Another route of administration that takes advantage of retrograde transport through neurons is intranasal administration. After introduction into the nasal cavity, molecules are believed to travel along olfactory nerve pathways and end up in the brain parenchyma and CSF by bypassing the BBB (Patel et al., 2009). Peptides, proteins, and small molecules have shown to be able to be delivered into the CNS after intranasal administration. While this route is non-invasive, the nasal cavity has many barriers including enzymes and mucous; furthermore, compounds similar to those that have shown CNS delivery have reportedly not entered the CNS after intranasal administration (Dhuria et al., 2010). The formulation and method of delivery may affect retrograde transport as well as other experimental factors such as head position, volume, pH and osmolarity (Dhuria et al., 2010). Plasmid DNA ranging from 3.5 kb to 14.2 kb were able to show absorption and brain distribution after intranasal administration (Han et al., 2007). Intranasal delivery of an telomerase-inhibiting oligonucleotide was able to prolong the survival of human tumor-bearing rats by over 30 days (Hashizume et al., 2008). Carriers for intranasal gene delivery follow the same principles as other vehicles administered by other routes. Polymeric vehicles comprised of methoxy PEG, poly(ɛ-caprolactone) and TAT peptide demonstrated better delivery of siRNA to the brain than naked siRNA or carrier-mediated intravenous delivery (Kanazawa et al., 2013).

## Conclusion

While viral vectors are still the main type of vehicles used in clinical trials, non-viral vectors are gaining traction due to their potential safety advantages, greater customization, ease of manufacturing and ability to deliver all nucleic acid varieties (Niidome and Huang, 2002; Thomas et al., 2003). The ability of viral vectors to permanently alter the genome and activate the immune system make non-viral vectors more compelling for clinical trials. However, the lower efficacy and transfection levels by synthetic vectors hinder their wide clinical use. For any application, nucleic acid delivery vehicles face cellular obstacles such as recognition by pertinent cells, internalization into cells, escaping the lysosomal degradation pathway and unpackaging the nucleic acids in the cell cytosol. Delivery into the CNS presents an even greater challenge due to the supracellular BBB and BSCB. An apropos administration route must be chosen to maximize therapeutics at the treatment site (i.e., direct injections); however, caution must be heeded in avoiding unnecessary damage to healthy tissues by direct administration into the CNS (i.e., intravenous and peripheral administration). Each route of administration has its advantages and disadvantages, as well as local barriers, as previously discussed. Fortunately, the advances in vehicular design, materials and synthesis described above have allowed for specific engineering of gene delivery vehicles to overcome these challenges and step closer to the transfection efficiency of viruses. Improvements such as nucleic acid shielding and targeting have lessened premature degradation and increased the localization of cargo in the CNS. Advances in crossing the BBB and sustained delivery directly into the brain allow for improved gene transfection and a step closer to clinical application. Meanwhile, new techniques such as microbubble-mediated sonoporation and small molecule-mediated retrograde transport allow the permeation of otherwise excluded vehicles into the brain and spinal cord. All of these examples can serve as guidelines and inspiration for the next generation of synthetic gene delivery vectors. With these improvements, we anticipate that synthetic delivery systems will be applied more successfully for nucleic acid therapies in animal models of CNS disease and will make significant progress toward clinical evaluation in the upcoming years.

## Author Contributions

J-KYT: led conception, writing of review, drafting and editing figures. SHP and PJH: conceived, assisted in writing and editing topic. BP and DLS: assisted in writing and editing topic.

## Conflict of Interest Statement

Authors have a US patent (61/532,982) regarding TAxI peptide.
